# Phase 3 multicenter randomized trial of PSMA PET/CT prior to definitive radiation therapy for unfavorable intermediate-risk or high-risk prostate cancer [PSMA dRT]: study protocol

**DOI:** 10.1186/s12885-021-08026-w

**Published:** 2021-05-07

**Authors:** Jeremie Calais, Shaojun Zhu, Nader Hirmas, Matthias Eiber, Boris Hadaschik, Martin Stuschke, Ken Herrmann, Johannes Czernin, Amar U. Kishan, Nicholas G. Nickols, David Elashoff, Wolfgang P. Fendler

**Affiliations:** 1 Ahmanson Translational Theranostics Division, Department of Molecular & Medical Pharmacology, David Geffen School of Medicine, University of California, Peter Norton Medical Building, 200 Medical Plaza, Suite B-114-51, Los Angeles, CA 90095-7370 USA; 2 Jonsson Comprehensive Cancer Center, University of California Los Angeles, Los Angeles, CA USA; 3 Institute of Urologic Oncology, University of California Los Angeles, Los Angeles, CA USA; 4 Department of Nuclear Medicine, University of Duisburg-Essen and German Cancer Consortium (DKTK)-University Hospital Essen, Hufelandstraße 55, 45131 Essen, Germany; 5 Department of Nuclear Medicine, Klinikum rechts der Isar, Technical University Munich, Munich, Germany; 6 Department of Urology, University of Duisburg-Essen and German Cancer Consortium (DKTK)-University Hospital Essen, Essen, Germany; 7 Department of Radiotherapy, University Hospital Essen, University of Duisburg-Essen, Essen, Germany; 8 Department of Radiation Oncology, David Geffen School of Medicine, University of California, Los Angeles, USA; 9 Department of Radiation Oncology, VA Greater Los Angeles Healthcare System, Los Angeles, California USA; 10 Department of Urology, David Geffen School of Medicine, University of California, Los Angeles, USA; 11 Department of Medicine Statistics Core (DOMStat), UCLA CTSI Biostatistics and Computational Biology, University of California, Los Angeles, USA

**Keywords:** Prostate cancer, PSMA, PET/CT, Randomized phase 3 trial, Definitive radiation therapy

## Abstract

**Background:**

Definitive radiation therapy (dRT) is an effective initial treatment of intermediate-risk (IR) and high-risk (HR) prostate cancer (PCa). PSMA PET/CT is superior to standard of care imaging (CT, MRI, bone scan) for detecting regional and distant metastatic PCa. PSMA PET/CT thus has the potential to guide patient selection and the planning for dRT and improve patient outcomes.

**Methods:**

This is a multicenter randomized phase 3 trial (NCT04457245). We will randomize 312 patients to proceed with standard dRT (control Arm, *n* = 150), or undergo a PSMA PET/CT scan at the study site (both 18F-DCFPyL and 68Ga-PSMA-11 can be used) prior to dRT planning (intervention arm, *n* = 162). dRT will be performed at the treating radiation oncologist facility. In the control arm, dRT will be performed as routinely planned. In the intervention arm, the treating radiation oncologist can incorporate PSMA PET/CT findings into the RT planning. Androgen deprivation therapy (ADT) is administered per discretion of the treating radiation oncologist and may be modified as a result of the PSMA PET/CT results. We assume that approximately 8% of subjects randomized to the PSMA PET arm will be found to have M1 disease and thus will be more appropriate candidates for long-term systemic or multimodal therapy, rather than curative intent dRT. PET M1 patients will thus not be included in the primary endpoint analysis. The primary endpoint is the success rate of patients with unfavorable IR and HR PCa after standard dRT versus PSMA PET-based dRT. Secondary Endpoints (whole cohort) include progression free survival (PFS), metastasis-free survival after initiation of RT, overall survival (OS), % of change in initial treatment intent and Safety.

**Discussion:**

This is the first randomized phase 3 prospective trial designed to determine whether PSMA PET/CT molecular imaging can improve outcomes in patients with PCa who receive dRT. In this trial the incorporation of PSMA PET/CT may improve the success rate of curative intent radiotherapy in two ways: to optimize patient selection as a biomarker and to personalizes the radiotherapy plan.

**Clinical trial registration:**

**UCLA**

- IND#147591
  - ○ Submission: 02.27.2020
  - ○ Safe-to-proceed letter issued by FDA: 04.01.2020
- UCLA IRB #20–000378
- ClinicalTrials.gov Identifier NCT04457245. Date of Registry: 07.07.2020.

**Essen**

- EudraCT 2020–003526-23

## Background

### Definitive Radiation Therapy for clinically localized prostate cancer

Standard options for the initial management of men with clinically localized prostate cancer (PCa) include radiation therapy (RT; external beam and/or brachytherapy, with or without androgen deprivation therapy [ADT]), radical prostatectomy, or active surveillance in carefully selected patients. The choice of treatment is determined by a variety of factors, including risk stratification, patient preference, clinician judgment, and resource availability. Although there are few randomized trials comparing RT with radical prostatectomy, the trials completed to date and observational data suggest that outcomes with either external beam RT or brachytherapy (using adequate dosing schedules and contemporary treatment techniques) are similar to those with radical prostatectomy when men with clinically localized PCa are stratified based on clinical tumor (T) stage, pretreatment serum prostate-specific antigen (PSA), and Gleason score [1–4]. Several studies have evaluated treatment outcomes for definitive radiation therapy (dRT) for PCa in patients with low-, intermediate- (IR) and high-risk (HR) disease. Progression-free survival (PFS) for patients with IR and HR PCa following dRT treatment ranges from 53 to 97% and 42–86%, respectively (Table 1) [5].

**Table 1:** Studies evaluating several treatment regimens of RT on patients with low-, intermediate- and high-risk PCa

Author, year	Ref	RT type	n	total dose (Gy)	total fractions	Gy/ fraction	PFS def.	Risk group n			5-year PFS (%)		
								LR	IR	HR	LR	IR	HR
Kuban, 2008	[6]	3D	151	78	39	2	P	30	68	53	100	86	69
		3D	150	70	35	2	P	31	71	48	88	83	54
Al-Mamgani, 2008	[7]	3D	333	78	39	2	P	63	90	180	N/A	70	N/A
		3D	331	68	34	2	P	56	89	185	N/A	60	N/A
Zietman, 2010	[8]	3D	195	79	44	1.8	P	116	72	7	95	79	N/A
		3D	197	70	39	1.8	P	111	76	10	75	68	N/A
Dearnaley, 2007	[9]	3D	422	74	37	2	P	99	127	184	71	71	N/A
		3D	421	64	32	2	P	95	137	175	60	60	N/A
Michalski, 2010, 2012	[10, 11]	3D	108	68	38	1.8	P	55	37	69	68	70	42
		3D	300	74	41	1.8	P	91	75	39	73	62	62
		3D	167	79	44	1.8	P	85	54	36	67	70	70
		3D	256	74	37	2	P	92	109	40	84	74	54
		3D	220	78	39	2	P	80	109	32	80	69	67
Beckendorf, 2011	[12]	3D	153	70	35	2	P	–	153	–	–	68	–
		3D	153	80	40	2	P	–	153	–	–	74	–
Michalski, 2014	[13]	3D+ IMRT	748	79	44	1.8	P	–	748	–	–	75	–
		3D+ IMRT	751	70	39	1.8	P	–	751	–	–	60	–
		3D	(491)	N/A	N/A	N/A	P	30	68	53	N/A	N/A	N/A
		IMRT	(257)	N/A	N/A	N/A	P	31	71	48	N/A	N/A	N/A
Lukka, 2005	[14]	3D	470	66	33	2	A	–	470	–	N/A	53	N/A
		3D	466	52	20	2.63	A	–	466	–	N/A	60	N/A
Yeoh, 2011	[15]	3D	109	64	32	2	P	–	109	–	N/A	58	N/A
		3D	108	55	20	2.75	P	–	108	–	N/A	69	N/A
Arcangeli, 2012	[16]	3D	85	80	40	2	P	–	–	85	N/A	N/A	79
		3D	83	62	20	3.1	P	–	–	83	N/A	N/A	85
Pollack, 2013	[17]	IMRT	152	76	38	2	P	–	101	51	N/A	86	86
		IMRT	151	70	26	2.7	P	–	98	53	N/A	86	86
Kuban, 2010	[18]	IMRT	102	76	42	1.8	P	30	1	1	96	96	N/A
		IMRT	102	72	30	2.4	P	30	1	1	97	97	N/A
Mantz, 2014	[19]	Gantry	102	40	5	8.0	P	40	–	–	100	N/A	N/A
Katz, 2010, 2011	[20, 21]	RA	304	35	5	7.0	P	211	81	12	99	93	75
				36	5	7.3	P						
Fuller, 2014	[22]	RA	60	38	4	9.5	P	40	39	–	100	92	N/A
Dubray, 2016	[23]	3D without ADT	191	80	40	2	–	–	191	–	–	76	–
		with ADT	179				–	–	179	–	–	84	–
Dong, 2017	[24]	3D/IMRT without ADT	979	74–80 or 70.2	40 or 26	1.8–2.7	–	–	979	–	–	87.3	–
		with ADT	155				–	–	155	–	–	84	–

*3D-CRT* 3 dimensional conformal radiation therapy, *PFS* progression-free survival, *HR* high risk, *IMRT* intensity modulated radiation therapy, *IR* intermediate risk, *LR* low risk, *N/A* not applicable. The definition of PFS (Phoenix, P; or ASTRO A) is listed

Conventional Imaging studies (radionuclide bone scan, computed tomography [CT] of the abdomen and pelvis, multiparametric magnetic resonance imaging [MRI]) are used selectively to assess for extraprostatic extension, regional lymphadenopathy, or distant metastases, for patients with IR and HR disease [25]. MRI is used for early detection of cancer and also often used for the purpose of assisting with RT planning and contouring..

Positron emission tomography (PET) using small molecule probes targeting prostate-specific membrane antigen (PSMA PET/CT) is superior to standard of care imaging for detecting regional and distant metastatic recurrent PCa at low PSA levels [26–30], highly specific [30] and reproducible [31]. Studies have demonstrated clear diagnostic accuracy superiority in large numbers of patients with biochemical recurrent PCa after curative treatment compared to conventional imaging for detection of locoregional recurrence and/or metastases [29, 32]. PSMA PET/CT outperformed planar bone scan for detection of osseous metastases in large retrospective analyses [33, 34]. The detection rate of PSMA PET/CT for recurrent PCa exceeds that of choline PET [35, 36], and of ^18^F-Fluciclovine PET [29, 37, 38]. PSMA PET/CT can improve RT planning and patient selection for salvage radiation therapy (SRT) thus may potentially improve its outcome [39–41]. Randomized trials investigating the outcome of SRT based on PSMA PET/CT and conventional imaging are now ongoing (NCT03525288, NCT03762759, NCT03582774) [42].

### Impact of PSMA PET/CT on primary staging of prostate Cancer

The potential role of PSMA PET/CT in *primary* staging of patients with IR and HR PCa has been explored in only a small number of studies outlined below (Table 2).

Despite the clear diagnostic superiority of PSMA PET/CT in initial staging of PCa [43], its impact on outcome of patients with IR and HR PCa has not been assessed prospectively. At the time of study design, several mostly retrospective studies reported the accuracy of PSMA PET/CT at initial staging (Table 2). Six studies (highlighted in **bold**) evaluated the impact of PSMA PET/CT (whether exclusively or not) on dRT planning of patients. Four of these studies are retrospective. One prospective study is a multicenter Australian study with 420 patients (108 patients for primary staging of IR and HR disease and 312 patients for restaging/biochemical recurrence) [44]. Comparison was made between both groups and the impact was shown to be greater in the group of patients with biochemical failure after definitive surgery or dRT than in patients undergoing primary staging. The other prospective study is a US post-hoc analysis of an intention-to-treat population of 73 patients with localized PCa without prior local therapy who underwent PSMA PET/CT for initial staging [45]. The scan had a major impact on intended definitive PCa dRT planning in 16.5% of patients when RT fields were intended to cover the prostate, seminal vesicles and the pelvic LNs, and in 37% when RT fields were intended to cover only the prostate and seminal vesicles. Recent studies from Koerber et al. detail aspects of nodal involvement and highlight impact in a mixed primary/recurrence population [46, 47].

**Table 2:** Literature review of the impact of PSMA PET on primary staging of patients with prostate cancer

Author and year	Study Design	Location	N	Population	Median PSA ng/mL (range)	Improvement with PSMA PET
Budäus et al. 2016 [48]	R	Hamburg, Germany	30	HR PCa prior to RP	8.8 (1.4–376)	Se 33%, spec 100%, PPV 100%, NPV 69%
**Calais et al. 2018** [45]	**P**	**Los Angeles, USA**	**73**	**IR/HR PCa prior to RT planning**	**13.9 (0.22–909)**	**9.5% uptaged to M1**
Demirkol et al. 2015 [49]	R	Istanbul, Turkey	8	HR PCa for staging	15 (0.3–20)	N/A
Fendler et al. 2016 [50]	R	Munich, Germany	21	PCa for staging	N/A	Se 67%, spec 92%, PPV 97%, NPV 42% Acc 72%
**Frenzel et al. 2018** [51]	**R**	**Hamburg, Germany**	**20**	**PCa prior to RT planning**	**7.1 (0.48–137)**	**N/A**
Herlemann et al. 2016 [52]	R	Munich, Germany	20	HR PCa prior to RP	^a^56 (3.3–363)	Se 84%, spec 82%, PPV 84%, NPV 82%
Hijazi et al. 2015 [53]	R	Göttingen, Germany	12	PCa for staging	48 (6–90)	Se 94%, spec 99%, PPV 89%, NPV 99.5%
Hirmas et al. 2018 [54]	R	Amman, Jordan	21	HR PCa for staging	38 (0.6- > 100)	Se 85% Acc 85.7%, PPV 100%
**Hruby et al. 2018** [55]	**R**	**NSW, Australia**	**109**	**IR/HR PCa prior to EBRT**	**9.9 (1.23–240)**	21% upstaged, 3% downstaged
Kabasakal et al. 2015 [56]	R	Istanbul, Turkey	15	PCa for staging	37.78 (5.12–70.47)	N/A
Maurer et al. 2015 [57]	R	Munich, Germany	130	HR PCa prior to RP	11.6 (0.57–244)	Se 68%, spec 99%, PPV 95%, NPV 94%
Rahbar et al. 2015 [58]	P	Münster, Germany	6	HR PCa prior to RP	52.7 (5.7–111.1)	Se 92%, spec 92%, PPV 96%, NPV 85%
Rhee et al. 2016 [59]	P	Queensland, Australia	20	PCa prior to RP	6.1 (3.5–45)	Se 49%, spec 95%, PPV 85% NPV 88%
**Roach et al. 2017** [44]	**P**	**Sydney, Australia**	**108**	**IR/HR PCa for staging**	**8.6 (0.18–120)**	**20% upstaged, 1% downstaged**
Sachpekidis et al. 2016 [60]	P	Heidelberg, Germany	24	HR PCa	24.1 (3.2–200)	N/A
Schwenck et al. 2016 [61]	P	Tübingen, Germany	20	HR PCa for staging, PSMA vs choline	26 (N/A)	N/A
**Sterzing et al. 2016** [62]	**R**	**Heidelberg, Germany**	**15**	**HR PCa for staging**	**7 (0.28–45)**	**13.7% changed their TNM staging**
Uprimny et al. 2017 [63]	R	Innsbruck, Austria	90	PCa, other analysis	9.7 (2.2–188.4)	N/A
Van Leeuwen et al. 2017 [64]	P	Sydney, Australia	30	IR/HR PCa prior to RP	8.1 (5.2–10.1)	Se 58%, spec 100%, PPV 94%, NPV 98%
**Zamboglou et al. 2015** [65]	**R**	**Freiburg, Germany**	**22**	**PCa prior to RT planning**	**20.4 (1.22–66.9)**	**GTV-PET larger than GTV-MRI**

^a^Value for mean reported, not median. *GTV* gross tumor volume, *HR* High-risk, *IR* intermediate-risk, *LND* lymph node dissection, *N/A* not applicable, *NPV* negative predictive value, *P* prospective study, *PPV* positive predictive value, *R* retrospective study, *RP* radical prostatectomy, *RT* radiotherapy, *Se* Sensitivity, *spec* specificity

PSMA PET/CT thus has the potential to guide dRT planning in patients and improve outcomes. Five studies have shown that dRT planning based on PSMA PET/CT results can change local plan in 13–19.5% and detect extra-pelvic disease in 6.4–9.5% of patients (Table 3).

**Table 3:** Changes in RT plan in studies assessing effect of PSMA PET results on treatment plan

Author and year	N	Inclusion criteria	Median PSA level, ng/mL (range)	Change in planned pelvic RT		
				% local plan change	% extra-pelvic disease	local plan change details
Calais et al. 2018 [54]	73	IR/HR PCa prior to RT planning	13.9 (0.22–909)	7–19.5%^a^	9.5%	covered pelvic LNs detected
Frenzel et al. 2018 [51]	20	PCa prior to RT planning	7.1 (0.48–137)	15%	N/A	shifted from IR to HR, one patient had boost to distant PET findings
Hruby et al. 2018 [55]	109	IR/HR PCa prior to RT planning	9.9 (1.23–240)	14.7%	6.4%	covered pelvic LNs detected
Roach et al. 2017 [44]	108	IR/HR PCa prior to RP/ EBRT/ systemic treatment	8.6 (0.18–120)	15%	9%	higher dose and volume
Sterzing et al. 2016 [62]	15	HR PCa for staging	7 (0.28–45)	13%	N/A	covered pelvic LNs detected

^a^Depending on initial intent to include elective pelvic nodal RT (change: 7%) or not (change: 19.5%). ^+^Mean value reported, not median. *EBRT* external-beam radiotherapy

### Treatment outcomes of dRT for PCa

Several studies have evaluated treatment outcomes of dRT for PCa in patients with low-, IR and HR disease. PFS for patients with IR and HR PCa following dRT ranges from 53 to 97% and 42–86%, respectively (Table 3) [5].

### Current prospective trials evaluating the impact of PSMA PET/CT on prostate RT planning

A prospective, randomized and multicenter study demonstrated superior accuracy of PSMA PET/CT along with impact on first- and second-line management of PCa patients with high-risk features (proPSMA study) [43]. Among the 339 recruited men, PSMA PET/CT-CT had a 27% greater accuracy than that of conventional imaging (92% vs 65%). There was higher rate of management change associated with PSMA PET/CT vs. conventional imaging (41 vs. 23% men).

Trial NCT03525288 currently taking place at Centre hospitalier de l’Université de Montréal (CHUM), Canada, aims to compare second generation ^18^F-DCFPyL PET with conventional imaging prior to RT planning in patients with HR, recurrent or oligometastatic PCa. Patients included will be randomized to either ^18^F-DCFPyL PET or conventional imaging prior to treatment planning, which will depend on imaging results of each arm. PFS will be assessed as primary endpoint with aims of showing that PSMA PET/CT findings will lead to improved cancer control outcomes compared to RT guided by conventional staging only.

Trial NCT03344822 taking place in Central Hospital in Nancy, France, aims to evaluate the difference of management intent after the initial staging of patients with HR PCa with PSMA PET/CT in comparison of ^18^F-Choline PET results. Each patient will receive a ^18^F-Choline PET followed by PSMA PET/CT.

It is unclear if incorporation of PSMA PET/CT imaging into the planning of dRT could improve its likelihood of success. There is no current randomized prospective trial designed to determine whether PSMA PET/CT can improve outcomes in patients with primary IR or HR PCa undergoing dRT.

The purpose of the present trial is to evaluate the success rate of is to compare the success rate of patients with unfavorable IR and HR PCa after standard dRT versus PSMA PET-based dRT.

#### Rationale for study design and hypothesis

**Fig. 1:**
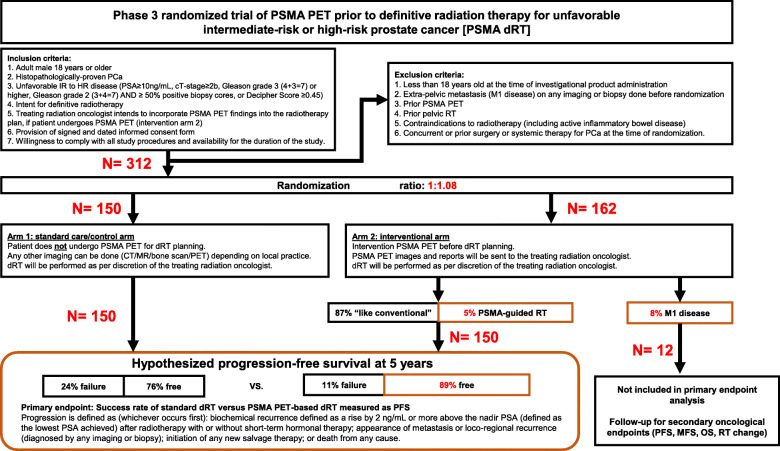
Flowchart of literature search and selection of studies

The overall study design is shown in Fig. 1

From our comprehensive literature review and subsequent meta-analysis, we have found that the 5-year PFS with dRT given to patients with primary PCa (IR and HR combined), with or without ADT, reaches 76% based on data from the meta-analysis of Table 1 studies (random effect-model). In addition, PSMA PET/CT changes the management plan prior to RT for patients with primary PCa in at least 13% of cases using the same model (meta-analysis of Table 3 studies). Therefore, we hypothesized that the incorporation of PSMA PET/CT to RT planning would improve the 5-year PFS of dRT in patients with unfavorable IR or HR PCa by 13% to reach 89% (intervention group) versus 76% without PSMA PET/CT (control group).

In this study, patients will be randomized into two arms:

1. standard RT arm 1: Patient does not undergo PSMA PET/CT for RT planning. RT will be performed as routinely planned in accordance to initial stratification. Any other imaging is allowed if done per routine care. If a control group patient undergoes a PSMA PET/CT scan at another institution he will be discontinued from the study.
2. PSMA PET/CT-based RT arm 2: Patient undergoes PSMA PET/CT for RT planning. Treating radiation oncologist will incorporate PSMA PET/CT findings into the RT planning and in accordance to initial stratification. Conventional imaging for staging purposes is not mandatory.

The 5-year PFS is expected to be 76% in Arm 1 (standard RT) and 89% in arm 2 (PSMA PET/CT-based RT), and since 8% will be found ineligible to continue this study post PSMA PET/CT, patients will be randomized in a 1:1.08 ratio. The primary endpoint of the trial is PFS. We will compare PFS between the two randomized treatment arms, stratified by very high risk (T3b or primary ISUP grade 5 pattern or ≥ 5 cores with International Society of Urological Pathologists (ISUP) grade group 4–5 or higher)) versus less than very high risk. We assumed, based on data from the meta-analyzed proportion for extra-pelvic disease (random effect model), that approximately 8% (*n* = 12) of subjects randomized to arm 2 will be found to be ineligible for dRT due to the presence of extra-pelvic metastatic (M1) detected by PSMA PET/CT (meta-analysis of Table 3 studies). These patients will not be included in analysis of the primary endpoint but will still be followed for other secondary endpoints.

The overall approach is to use PSMA PET not as a diagnostic tool but rather as biomarker for patient selection. The primary objective of the trial is not to assess whether curative RT will improve the oncological outcome of patients after PSMA-based staging. Rather, it is to determine if integration of PSMA PET/CT at the time of RT planning increases success rate of curative intent RT. We will compare the success rate of patients with unfavorable IR and HR PCa who actually underwent dRT: standard dRT versus PSMA PET-based dRT.

#### Objective of the trial

The aim of the study is to compare the outcome of patients with unfavorable IR and HR PCa after standard dRT versus PSMA PET/CT-based dRT. Outcome will be assessed in parallel trials at UCLA (^18^F-DCFPyL PET) and Essen (^68^Ga-PSMA-11 PET).

#### Trial design

This is a prospective multicenter interventional controlled randomized open-label phase 3 clinical trial with parallel assignment. The intervention is one PSMA PET/CT scan (both 18F-DCFPyL and 68Ga-PSMA-11 can be used) performed at the nuclear medicine department of the study site. DRT is performed at the treating radiation oncologist facility. Patients are followed remotely by the nuclear medicine clinical research team. The aim of the study is to compare the outcome of patients with unfavorable IR and HR PCa after standard dRT versus PSMA PET/CT-based dRT. Final analysis will be conducted on joint datasets.

A total of 312 patients will be randomized into two arms (randomization ratio 1:1.08): control arm 1 (*n* = 150, without PSMA PET/CT scan) and intervention arm 2 (*n* = 162 with PSMA PET/CT scan). Screening, randomization, follow-up and data management will be performed centrally. RT can be done anywhere, at the institution of choice of the referring radiation oncologist and/or the patient. The investigators will rely on the medical records obtained from the treating physicians as the primary source of outcome data.

The study is powered for 5-year PFS. If a patient assigned to the control arm undergo a PSMA PET/CT scan at another institution prior to dRT, he will be discontinued from the study. Trials at UCLA and Essen are independent. Final analysis may be conducted on joint datasets from multiple sites, if acquired under similar PSMA-targeted PET dRT randomized study protocols.

## Methods

### Study population

In order to be eligible to participate in this study, an individual must meet all of the following criteria:

1. Adult male 18 years or older.
2. Histopathologically-proven PCa
3. Unfavorable IR to HR disease
  - PSA ≥ 10 ng/mL
  - or cT-stage≥2b
  - or ISUP grade 3 (Gleason 4 + 3 = 7) or higher
  - or ISUP grade 2 (Gleason 3 + 4 = 7) AND ≥ 50% positive biopsy cores
  - or Decipher Score ≥ 0.45
4. Intent for definitive radiotherapy
5. Treating radiation oncologist intends to incorporate PSMA PET/CT findings into the radiotherapy plan, if patient undergoes PSMA PET/CT (intervention arm 2)
6. Provision of signed and dated informed consent form
7. Stated willingness to comply with all study procedures and availability for the duration of the study.

An individual who meets any of the following criteria will be excluded from participation in this study:

1. Less than 18 years old at the time of investigational product administration
2. Extra-pelvic metastasis (M1 disease) on any imaging or biopsy done before randomization
3. Prior PSMA PET/CT
4. Prior pelvic RT
5. Contraindications to radiotherapy (including active inflammatory bowel disease)
6. Concurrent or prior surgery or systemic therapy for PCa at the time of randomization.

### Intervention

#### Study procedure

Patients allocated to the PSMA dRT arm (arm 2) will undergo one PSMA PET/CT scan at the nuclear medicine department of the study site before dRT planning.

#### Investigational PET imaging drug

Small radiolabeled ligands ^18^F-DCFPyL (UCLA) or ^68^GaPSMA11 (Essen) can be used as the PET radiopharmaceutical.

#### PET/CT imaging protocol specifics

1. Oral hydration is recommended immediately after injection the radiotracer before start of the scan.
2. PET/CT images will be acquired at 50–100 min (target 60 min) after intravenous injection of the radiotracer.
3. PET/CT scan coverage will extend from mid-thigh to the vertex.
4. A diagnostic CT will be acquired just before the PET imaging acquisition for attenuation correction.
5. CT-Contrast may be administered if requested by the referring clinician or the attending nuclear medicine physician. Details are outside the scope of this study protocol.
6. PET images will be acquired in 3D mode with a weight-based time per-bed-position
7. The PET emission scan will be corrected for decay, dead-time, random events, and scatter. PET images will be corrected for attenuation using segmented attenuation data of the low-dose CT scan. PET images will be reconstructed using ordered subset expectation maximization (OSEM) and filtered to a spatial resolution of 5 mm (isotropic) with a Gauss-filter.

#### PET/CT imaging analysis and image transfer

PET/CT Images will be reviewed and analyzed using dedicated workstations by a board certified nuclear medicine physician and a board certified radiologist during consensus clinical readouts in the Nuclear Medicine Departments using recent reporting guidelines (PROMISE criteria, miTNM standardized framework) with access to all medical records [66]. No blinded independent central readers will be used.

CD/DVD containing the PSMA PET/CT DICOM images and PET/CT report will be systematically delivered to the treating radiation oncologist.

#### Radiation therapy (RT) management

DRT is performed at the treating radiation oncologist facility. The modality, dose, fractionation, and target volumes of the RT are at the discretion of the treating radiation oncologist. EBRT regimens including conventional, hypofractionated, and extremely hypofractionated (SBRT) are allowable. Brachytherapy (LDR or HDR), alone or in combination with EBRT, is also allowable. The use of neoadjuvant, concurrent, or adjuvant ADT is also at the discretion of the treating physician.

Routine care dRT for intermediate or HR localized PCa typically involves either radiotherapy directed to the prostate and seminal vesicles alone, or the prostate, seminal vesicles, and pelvic lymph nodes. In addition, the RT can be delivered either with or without concurrent ADT. The choice of treating the prostate and seminal vesicles alone with or without ADT vs including also pelvic lymph nodes with ADT, is based upon clinico-pathologic features and practice patterns of the treating physician. The treating physician will be asked to describe their general treatment plan prior to randomization.

In some cases, due to specific anatomical features of the patient (for example, location of small bowel), or patient’s preference (for example, patient may decline ADT), the general treatment plan may be modified during the radiotherapy planning process. The treating physician is encouraged not to de-escalate therapy based on results of the PSMA PET/CT. For example, a PSMA PET/CT with no evidence of disease outside the prostate does not exclude the possibility of microscopic disease outside the prostate (for example, in pelvic lymph nodes).

Patients randomized to arm 1 do not undergo PSMA PET/CT and dRT will be performed as routinely planned per discretion of the treating radiation oncologist in accordance with the initial general treatment plan whenever possible. Any other imaging is allowed for RT planning if done per routine care. Systemic therapy, if needed, will be performed as per discretion of the treating radiation oncologist or other physician. If a control group patient undergoes a PSMA PET/CT scan at another institution prior to dRT, he will be discontinued from the study.

Patients randomized to arm 2 that have a negative PSMA PET/CT scan will undergo RT as routinely planned by the treating radiation oncologist in accordance with the initial general treatment plan whenever possible. Concurrent systemic therapy, if needed, will be performed as per discretion of the treating radiation oncologist or physician.

Patients randomized to arm 2 where PSMA PET/CT detects PSMA-positive lesions ***within*** *the pelvis (prostate, seminal vesicles, extraprostatic extension, lymph nodes)* will undergo RT performed by the treating radiation oncologist in accordance with the new findings. For example, the plan may include adapted/extended target volumes to cover all pelvic PSMA-positive lesions within the irradiated volumes. Additionally, the RT may incorporate focal dose escalation to the PSMA-positive lesions. Concurrent systemic therapy, if indicated, will be performed as per discretion of the treating radiation oncologist or other physician, and may be escalated in intensity or duration as a result of the PSMA PET/CT findings (for example, due to discovery of N1 disease).

Patients randomized to arm 2 that have a PSMA PET/CT showing PSMA-positive lesions ***outside*** the pelvis or osseous structures within the pelvis (i.e., M1 disease) will undergo treatment as per discretion of the treating radiation oncologist or other physician. However, the patient will not be included in analysis of the primary endpoint, because curative intent dRT is not an option for de novo M1 patients. We assume that approximately 8% of subjects randomized to arm 2 will be found to be ineligible for RT due to PSMA detected M1, and thus will not be included for primary endpoint analysis.

### Outcome measures

#### Primary Endpoint Measures

Success rate of patients with unfavorable IR and HR PCa after standard dRT versus PSMA PET-based dRT measured as PFS after initiation of dRT [Time Frame: from date of randomization to first occurrence of progression, assessed up to 5 years]. Patients who do not underdo dRT are not included in the primary endpoint analysis.

Progression is defined as (whichever occurs first):

- A biochemical recurrence defined as a rise by 2 ng/mL or more above the nadir PSA (defined as the lowest PSA achieved) after radiotherapy with or without short-term hormonal therapy [67].
- Appearance of metastasis or loco-regional recurrence (diagnosed by any imaging or biopsy).
- Initiation of any new salvage therapy
- Death from any cause.

The investigators will rely on the medical records obtained from the treating physicians as the primary source of outcome data.

#### Secondary endpoints measures

1. Loco-regional progression free survival (PFS)

- Diagnosis of local recurrence or pelvic nodal disease (N1) can be obtained by any imaging or biopsy.
- Death from any cause

2) Metastasis-free survival after initiation of RT

- Diagnosis of extra-pelvic metastatic (M1) disease can be obtained by any imaging or biopsy.
- Death from any cause

3) Overall survival (OS)
4) Change in initial treatment intent (%) as assessed by the comparison of the intended RT plan collected on questionnaires before randomization to the delivered RT plan.
5) PSMA PET/CT derived predictors of PFS and OS.
6) Safety

The investigators will rely on questionnaire, study and medical records obtained from the treating physicians as the primary source of outcome data.

### Timeline

#### Screening and enrollment

Patients seen in consultation in a radiation oncology, urology/uro-oncology, or nuclear medicine clinic who are being evaluated for potential RT for PCa will be informed of this clinical study if eligible. Referring physicians will be educated on the study goals and logistics in order to recruit potential eligible and interested patients. The decision to participate will be entirely voluntary. Eligible patients who decide not to participate will be offered all other standard of care approaches. Treating radiation oncologist must intend to incorporate PSMA PET/CT findings into the radiotherapy plan if patient undergoes PSMA PET/CT (as per stratification). Patients will be consented either in person or over the phone (Signed consent form will be obtained by fax or email in the latter case).

#### Randomization and intervention

All the data management such as the randomization allocation will be performed by the lead institution in the online clinical trial database. This is an open label study. Trial participants, care providers, outcome assessors, and data analysts will be aware of the assignment after enrollment is completed. The randomization number and assignment will be communicated by phone or email to the treating physician. Patients will be informed by phone or email of the randomization assignment. If patient is randomized to investigational arm 2, he will be contacted and scheduled for a PSMA PET/CT scan at participating sites.

#### Outcome follow-up

Clinical, PSA and imaging follow-up will be conducted as per standard of care. Current standard of care usually includes weekly on treatment visits during radiotherapy followed by follow-up visits with radiation oncologist at least every 3 to 4 months for the first year and every 6 months for the next 5 years and annually thereafter (NCCN and ASTRO clinical guidelines). Imaging follow-up can be ordered when disease progression is suspected. We recommend restaging be initiated after PSA biochemical failure (Phoenix criteria). NCCN clinical guidelines recommend that work-up for progression may include prostate MRI, TRUS biopsy, abdominal CT, MRI, bone scan and/or PET. Interpretation of follow-up imaging will be performed by local reads.

Research investigators or their staff will conduct telephone and secure electronic messaging follow-ups with treating physicians at 3-month intervals for the first year following completion of radiotherapy, and then at 6 month intervals. Through these telephone and secure electronic messaging follow-ups with treating physicians, the study team can access PSA measurements and required follow-up information.

#### Study duration

We expect to enroll 312 patients within 2 years of study initiation. Patients will be followed (phone calls/ secure emails) until either one of the following conditions occur:

1. Five [5] years after the date of randomization.
2. Biochemical progression.
3. Diagnostic of metastatic disease.
4. Initiation of any additional salvage therapy.
5. Patient randomized to control arm 1 (control) undergo a PSMA PET/CT scan at another institution
6. Death.

### Sample size determination

From our comprehensive literature review and subsequent meta-analysis, we found that 13% of patients would have had at least one lesion detected by PSMA PET/CT that would otherwise not be covered by the standard radiation fields covering both the prostate and pelvic lymph nodes (RTOG consensus delineations, meta-analysis of 1 studies). We therefore hypothesize that the incorporation of PSMA PET/CT to RT planning will improve 5-year PFS by 13%. Based on our meta-analysis of Table 3. Studies (random effects model), we estimate that the 5-year PFS of patients with PCa (IR and HR combined), with or without ADT, after standard dRT would be 76%. Therefore, we assume the 5-year PFS to be 76% in arm 1 (standard dRT) and 89% in arm 2 (PSMA PET/CT-based RT). We also assume that approximately 8% of subjects randomized to arm 2 will have extra-pelvic metastasis detected by PSMA PET/CT, and therefore are not curable by dRT. These patients will therefore not be included in the analysis of the primary endpoint but will still be followed for other secondary endpoints. We will compare the PFS between the two randomized treatment arms, stratified by very high risk (T3b or primary ISUP grade 5 pattern or ≥ 5 cores with ISUP grade group 4–5 vs less than very high risk. Final analysis may be conducted on joint datasets from multiple sites, if acquired under similar PSMA-targeted PET dRT randomized study protocols.

When the sample size in each group is 138, with a total number of events required of 43, a 0.050 level two-sided log-rank test for equality of survival curves will have 80% power to detect the difference between a PSMA group freedom from failure rate at 5-years of 89% and a control group freedom from failure rate at 5-years of 76% (a constant hazard ratio of 2.35). To account for an approximate 5–10% drop-out rate, we will plan for 150 patients per group, requiring 312 patients randomized as we expect that 12 in the PSMA group will have M1 disease and not be evaluable for the primary endpoint. Therefore, we will study 312 patients that are planned for dRT.

In case the above estimated total number of events is not reached at the end of the prespecified follow-up period, extension of this period with respective additional funding will be requested.

### Allocation sequence generation, concealment mechanism and implementation

The randomization will be processed via Research Electronic Data Capture (REDCap) code for all subjects from all participating sites. The participating site(s) will receive the randomization assignment by the leading institution after enrollment. The list will only be accessible for researchers or study personnel not actively involved in the recruitment process. We will use one stratification factor: risk level (very high risk vs less than very high risk).

This is an open label study. Trial participants, care providers, outcome assessors, and data analysts will be aware of the assignment after enrollment is completed. The randomization number and assignment will be communicated to the treating physician and patient.

### Data collection, management and monitoring

The REDCap® study database will have validated range checks for data entry fields, branching logic, and rigorous pre-testing to make sure the data are appropriately capture. The nuclear medicine research team will enter all data of each patient into REDCap® database. The nuclear medicine research team will have full access to all interim and final results of the study through the REDCap® database and is responsible for the final decision to terminate the trial. There is no planned interim analysis. All the data management will be performed by the nuclear medicine research team in the REDCap® online database. During the clinical investigation, the UCLA nuclear medicine research team will evaluate the progress of the trial, including periodic assessments of data quality and timeliness, participant recruitment, accrual and retention, participant risk versus benefit, and other factors that can affect study outcome. All the datasets generated during the current study will be stored and managed on the REDCap® database. All data generated and/or analyzed during this study will be publicly available (own DOI) after completion of the study and the publication of the article of the final analysis of study. Even if the required number of patients to reach statistical power is not met, patients already enrolled in the trial will still be followed for 5 years as this would remain highly valuable and unique data.

### Statistical methods

- *Per-Protocol Analysis Dataset: n = 300 (*sample size in each group is *n* = 150)

The primary endpoint (5-year PFS) will be analyzed in patients who actually underwent dRT. We assumed that approximately 8% of subjects randomized to arm 2 will have extra-pelvic metastasis detected by PSMA PET/CT, and therefore will not be treated with dRT. These patients will not be included in the analysis of the primary endpoint but will still be followed for other secondary endpoints.

- *Intention-to-Treat (ITT) Analysis Dataset: n = 312 (all randomized participants)*

The secondary endpoints will be analyzed in all randomized participants.

- *Safety Analysis Dataset: n = 162 (intervention arm 2, PSMA PET/CT scan)*

Analysis of the primary endpoint will be conducted upon reaching a sample size in each group of at least 150 and total number of 43 events. Survival curves will be constructed using the Kaplan-Meier method. We will use a stratified log rank test in our primary analysis to compare the PFS between the two randomized treatment arms, stratified by very high risk (T3b or primary ISUP grade 5 pattern or ≥ 5 cores with ISUP grade group 4–5 vs less than very high risk. Secondary analyses will utilize Cox-proportional hazards regression models. These models will include covariates pelvic LN RT, ISUP grade, T stage, initial PSA, and PSMA PET/CT nodal stage. Residual analyses will be performed to evaluate the proportional hazards assumptions of the Cox model. We will estimate the 95% confidence interval for the restricted mean PFS (over the planned 5-year follow-up duration) in each treatment group as well as the 95% confidence interval for the difference in mean PFS between the groups.

Final analysis may be conducted on joint datasets from multiple sites, when acquired under similar PSMA-targeted PET dRT randomized study protocols.

Similar to the primary endpoint, secondary survival endpoints (loco-regional PFS, metastasis free survival after RT, OS) will be compared between groups with stratified log rank tests and then with Cox proportional hazards regression models to control for above baseline subject characteristics.

## Discussion

PSMA PET/CT is a highly sensitive imaging test to localize PCa. However, it is unclear if incorporation of PSMA PET/CT imaging into the planning of dRT could improve its likelihood of success. No randomized prospective trial has been designed to determine whether PSMA PET/CT can improve 5-year outcomes in patients with IR and HR PCa. The purpose of this trial is to compare the success rate of patients with unfavorable IR and HR PCa after standard dRT versus PSMA PET-based dRT.

The planned exclusion of patients with M1 disease identified by PET from the interventional arm for the primary endpoint may generate some confusion. The role of PSMA PET/CT as localization and stratification tool has been discussed extensively when building the study design by the investigators.

The primary endpoint is not PFS after randomization, rather, the primary endpoint is the success rate of curative intent radiotherapy (measured as PFS) in patients who undergo curative intent radiotherapy. To undergo curative intent radiotherapy, a patient must be eligible for it, and this eligibility is contingent upon having no M1 lesions on diagnostic imaging. We contend that it is ethically questionable to ignore PSMA PET/CT findings for choice of primary treatment, even if performed on trial.

In this trial the incorporation of PSMA PET/CT may improve the success rate of curative intent radiotherapy in two ways. First, PSMA PET/CT may identify patients who are found to have distant metastatic disease for whom curative intend radical radiotherapy may not be appropriate (other strategies would be more appropriate). Here, the PSMA PET/CT is used as a biomarker to optimize patient selection. A similar approach would be HER2 and Trastuzumab in breast cancer. The HER2 testing does not improve the outcome of all patients but identify patients HER2+ who will benefit from Trastuzumab. Second, in patients without distant metastatic disease, PSMA PET/CT may improve the efficacy of radiotherapy directed to local and regional disease (i.e., through more accurate target delineation and higher radiation dose delivered to gross disease). Here, PSMA PET/CT personalizes the radiotherapy plan for the patient based on the specific extent of their disease, rather than applying an essentially standardized set of treatment volumes and prescription doses.

The estimated 8% of patients randomized to arm 2 will be patients who are not eligible to receive curative intent radiotherapy. Our trial tests the clinical impact of individualized therapy provided by PSMA PET/CT. This can be considered within the broader framework of precision oncology. More commonly in the context of precision oncology, a genomic biomarker is used to guide patient selection for a systemic therapy appropriate only for biomarker selected patients. In our trial, a functional imaging biomarker is used to select patients for an anatomically defined local therapy. Genomic analyses and imaging represent two distinct sources of information. This biologic and anatomic data can be both prognostic of outcome and predictive for treatment selection. The common objective for both genomics-guided precision oncology and functional imaging-guided precision radiotherapy is the same: to match the right treatments to the right patients to improve efficacy of treatment and to avoid futile care.

The risk level of a PSMA PET imaging scan is insignificant, especially in patients who receive RT (at least 10,000 times the amount of radiation). However, the radiation oncologists may use the PSMA PET imaging scan to direct radiotherapy with the potential to modify RT parameters or exclude patients from RT. Thus, the study is considered “greater than minimal risk”. Even if the positive predictive value of PSMA PET is high (> 85%) [32, 68] we cannot formally rule-out treatment changes induced by false-positive findings. We also believe that it is important to know wether incorporating PSMA PET info does not improve cure rate because of false positives.

Potential pitfalls in study design include i) drop-out of patients randomized to the control arm as patients may be able to undergo PSMA PET/CT scans in other institutions; iv) potential FDA approval and incorporation into guidelines of PSMA PET/CT imaging probes (^68^Ga-PSMA-11 or ^18^F-DCFPyL) in the near future, which would in essence lead to termination of the outlined enrollment design. As PSMA PET/CT imaging may become standard of care, randomizing patients to the control arm would no longer be feasible. Therefore, the time period for patient recruitment may be limited. Even if the required number of patients to reach statistical power (*n* = 312) is not met, patients already enrolled in the trial will still be followed for 5 years as this would remain highly valuable and unique data.

This is the first prospective multicenter randomized phase 3 trial designed to determine whether PSMA PET/CT molecular imaging can improve outcome of dRT in patients with IR and HR PCa. Positive outcome would enable better patient selection, an important step towards individualized medicine.

## Data Availability

The nuclear medicine research team will have full access to all interim and final results of the study through the REDCap® database. There is no planned interim analysis. All data generated and/or analyzed during this study will be publicly available (own DOI) after completion of the study and the publication of the article of the final analysis of study. The datasets generated and/or analyzed during the trail will not be publicly available before completion of the study but can be available from the corresponding author on reasonable request.

## Abbreviations

68Ga-: Gallium-68
ADT: Androgen deprivation therapy
ASTRO: American Society for Radiation Oncology
CT: Computed tomography
DKTA: University of Duisburg-Essen and German Cancer Consortium
dRT: Definitive Radiation Therapy
FDA: Food and Drug Administration
GCP: Good Clinical Practice
HIPAA: Health Information Portability and Accountability Act
HR: High Risk
IND: Investigational New Drug
IR: Intermediate Risk
IRB: Institutional Review Board
ITT: Intention-To-Treat
MBq: MegaBequerel
LN: Lymph Node
mCi: millicurie
MRI: Magnetic resonance imaging
NCCN: National Comprehensive Cancer Network
OS: Overall Survival
OSEM: Ordered Subset Expectation Maximization
PCa: Prostate cancer
PET/CT: Positron Emission Tomography/Computed Tomography
PFS: Progression-free survival
PSA: Prostate-specific antigen
PSMA: Prostate-specific membrane antigen
REDCap: Research Electronic Data Capture
RT: Radiation Therapy
RTOG: Radiation Therapy Oncology Group
SBRT: Stereotactic Body Radiation Therapy
TRUS: Transrectal Ultrasound
UCLA: University of California, Los Angeles
US: United States
